# Posttranslational Processing and Modification of Cathepsins and Cystatins

**DOI:** 10.1155/2010/375345

**Published:** 2010-12-16

**Authors:** Nobuhiko Katunuma

**Affiliations:** Institute for Health Sciences, Tokushima Bunri University, 180 Nishihamabouji, Yamashiro-cho, Tokushima City, Tokushima 770-8514, Japan

## Abstract

Cathepsins are an essential protease family in all living cells. The cathepsins play an essential roles such as protein catabolism and protein synthesis. To targeting to various organella and to regulate their activity, the post translational-processing and modification play an important role Cathepsins are translated in polysome as the pre-pro-mature forms. The pre-peptide is removed cotranslationally and then translocated to Golgi-apparatus and the pro-part is removed and the mature-part is glycosylated, and the mature-part is targeted into the lysosome mediated by mannose-6-phosphate signal and the mature-part is bound with their coenzymes. The degradation of the mature-part is started by the limited proteolysis of the ordered nicked bonds to make hydrophobic peptides. The peptides are incorporated into phagosome or proteasome after ubiquitinated and are degrade into amino-acids. Cystatins are endogenous inhibitors of cathepsins. Cystatin **α** which is only located in skin is phosphorylated at the near C-terminus by protein kinase-C, and the phosphorylate-cystatin **α** is incorporated into cornified envelope and conjugated with filaggrin-fiber by transglutaminase to form the linker-fiber of skin. The cystatin **α** is modified by glutathione or make their dimmer, and they are inactive. Those modifications are regulated by the redox-potential by the glutathione.

## 1. Introduction

**Figure 1:**
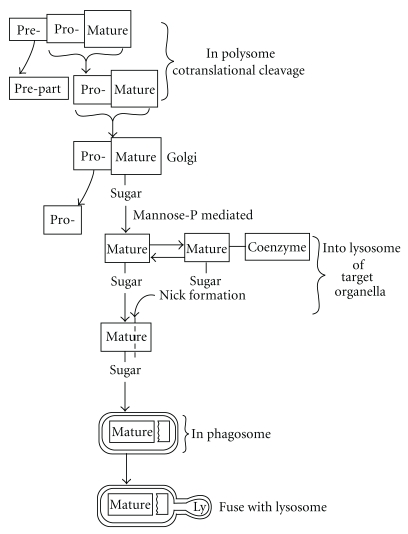
Common process of post-translational proteins in general.

We will introduce recent advances on the study of post-translational processing, modification, and targeting of cathepsins and cystatins. Almost all the intracellular proteins are passed through principally similar processes from the synthesis to their degradation in general. Therefore, I would like to introduce the general fate of intracellular proteins, from the post-translational processing, modification, and targeting to the ordered particles. As Figure 1 shows, the intracellular proteins are synthesized as pre-promature complex in polysomes and prepart is removed cotranslationally, and then the promature parts are translocated into Golgi-apparatus, and then glycosylated by mannose-rich sugar. The glycosylated mature part is translocated into target organelles and the degradation was started by the splitting from the ordered nicked bonds to make hydrophobic peptides. These hydrophobic peptides are secreted to cytoplasm and are incorporated into the phagosomes or proteosomes after ubiquitination. 

Biological merit of post-translational processing [1] and modifications of proteins are Possible considerations. The capability to take variable forms on the way of biosynthesis is important to keep adaptability to the changing of biological requirements and intracellular translocation during the maturation must be regulated. Active enzyme amount must be regulatable. Pro-parts or bound sugar are the targeting signals in some cases. In the cases of carbamoyl phosphate synthetase (CPS) and ornithine transcarbamylase (OTC), their pro-parts play the role of signals to be recognized by their receptors located on target organella membrane, such as lysosomes, as shown in Figure 4.

The glycosylated cathepsins are targeted into lysosomes mediated by mannose-6 phosphate receptors which are located on the lysosomal.

## 2. Cathepsins

### 2.1. Cathepsins, such as B, H, and L Are Located in the Different Lysosomes [2, 3]

As Figures 4(a) and 4(b) show, The lysosomes in which cathepsin H or B is located are attached to the cell-membrane. On the contrary, cathepsin L is located in the lysosomes which are distributed diffusely in liver cells. As Figure 4(a) shows, using immunodouble gold-particle staining, cathepsin B (small gold particle) and cathepsin H (large gold particle) are located clearly in different lysosomes [4]. These different localizations are important aspect of functional share of the different cathepsins.

### 2.2. Post-Translational Processing and Maturation of Cathepsins L, B, and H [5]

**Figure 2:**
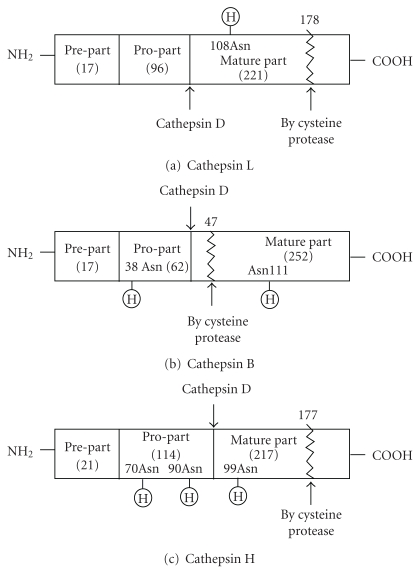
Post-translational processing and modification of cathepsin L, B, and H.

**Figure 4:**
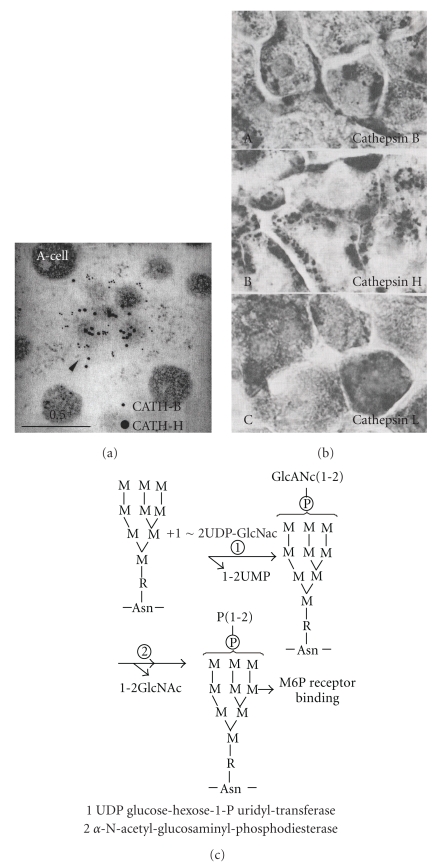
Characteristic localization in hepatocytes of cathepsins B, H, and L. (Rat liver) (a) Immuno-gold-particle staining of cathepsin B and H. (b) From head to down order, B, H, and L. (c) Cathepsin bound mannose-rich sugar compositions. Binding to M6P-Receptor binding.

Post-translational processing and maturation are summarized in Figure 2 [6–8].

Cathepsin L is translated as 17 amino acids of prepart, 96 amino acids of pro-part and 221 amino acids of mature-part [9, 10]. The prepart is removed cotranslationally and formed procathepsin is translocated into Golgi-apparatus, and then the 108-Asn and the 155-Asn in mature parts are glycosylated by high-mannose-type sugar, as shown in Figure 2(a). The initiation of the degradation is started from the nicking bond (178 bond) cleavage by some cysteine protease [7].

Cathepsin B [11] is translated as 17 amino acids of prepart, 62 amino acids of pro-part, and 252 amino acids of mature part [12, 13]. The prepart is also removed cotranslationally and the formed procathepsins are translocated into Golgi-apparatus and then the 38th-Asn in pro part and the 111th-Asn in the mature-part are glycosylated by high-mannose-type sugar.

Then the mannose-6-phosphate-moities play a role as the targeting marker to the lysosomes. The pro-part was cleaved off to make mature cathepsins in the lysosome. The cleavage ordered nicking at 47th bond was starting to be degraded.

Cathepsin H is translated as 21 amino acids of prepart [14], 114 amino acids of pro-part, and 217 amino acids of mature part. The pro-part has two carbohydrate chains at the 70th-Asn and the 90th-Asn and the mature part consisted of 217 amino acids, and one carbohydrate chain is bound at the 99th-Asn [8, 15]. The initiation of degradation is started from the 177th nicking bond by cysteine protease.

### 2.3. Turnover of Cathepsins [17]

Intracellular protein degradation (= autophagosome formation) is regulated by nutritional and hormonal conditions. Fasting or insulin enrichment caused stimulation of the phagosome formation and cathepsin L amount are increased; on the contrary, refeeding and glucagon enrichment resulted in the suppression of phagosome formation and decreasing of cathepsin L amount.

As you see in Figure 2, cathepsins B, L, and H have individual ordered nicking bond as the initiation of their degradations; these bonds are cleaved by cysteine protease, therefore as Table 1 shows, the half-lives (*t*_1/2_) of cathepsins and the contents in lysosomes clearly increased by treatment by E-64 (inhibitor for all cysteine proteases).

The initiations of degradation of cathepsins are started from the ordered limited proteolysis by cysteine protease in the lysosomes, as Figure 2 shows. The formed hydrophobic products were translocated into autophagosome or proteasome (after ubiquitinated) to degrade to amino acids.

### 2.4. Secretion of Cathepsins and Pathogenesis of the Secreted Cathepsins

Procathepsins or mature cathepsins are secreted from the various cells and play individual physiological, or pathological roles.

**Figure 5:**
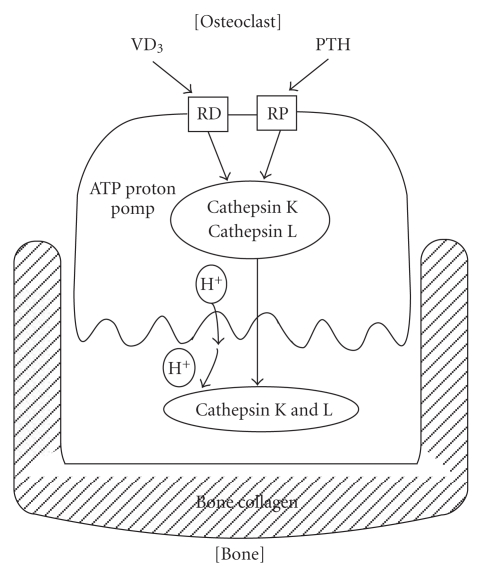
Bone resorption by osteoclastic cells, roles of secreted cathepsin K, and K mediated by ATP proton pomp.

As Figure 5 shows, bone metabolism is consisted of functional balances between osteoblastic cell function and osteoclastic cell function.

Bone collagen is degraded mainly by cathepsin L and K, therefore they are called collagenolytic cathepsins. The secretion of these cathepsins from osteoclasts is stimulated by PHT (Parathyroid Hormone) and suppressed by vitamin D_3_, E-64, CA-148 eta. Therefore, bone metastasis of cancers is inhibited by cathepsin inhibitors, such as by E-64 or CLIK-148 [18].

As Pit formation test in Table 4 shows, bone-matrix degradation is catalyzed by cathepsin L, but not by cathepsin B.

## 3. Cystatins

Endogenous low-molecular weight cathepsin inhibitors are consisted of two big groups [19], one is only located in skin and the other is ubiquitously located in all organs [16]. Cystatin *α* (A) [20] is only located in epidermis, cystatin *β* (B) [21] is located ubiquitously in all cells (organs) [22]. The secretary formed cystatins are Cystatin C in eggwhite and cystatin S in the saliva. *γ*-trace is secreted from cerebrospinal fluid. Another secretary group having high-molecular weight (repeated low molecular cystatins) is kininogen family in serum [22].

Each cystatin shows different inhibitory specificity for individual cathepsins, for example, cystatin *α* does not inhibit cathepsin B activity but strongly inhibit cathepsin L activity and cathepsin H activities [7, 19]. This is important as their function shares against cathepsin family.

### 3.1. Post-Translational Processing of Cystatins

The amino-acid sequences of low-molecular weight cystatin family have strong homology, however, their post-translational processing are quite different. Because their localizations and biological functions is quite different.

#### 3.1.1. Cystatin *α* [23–25]

**Figure 7:**
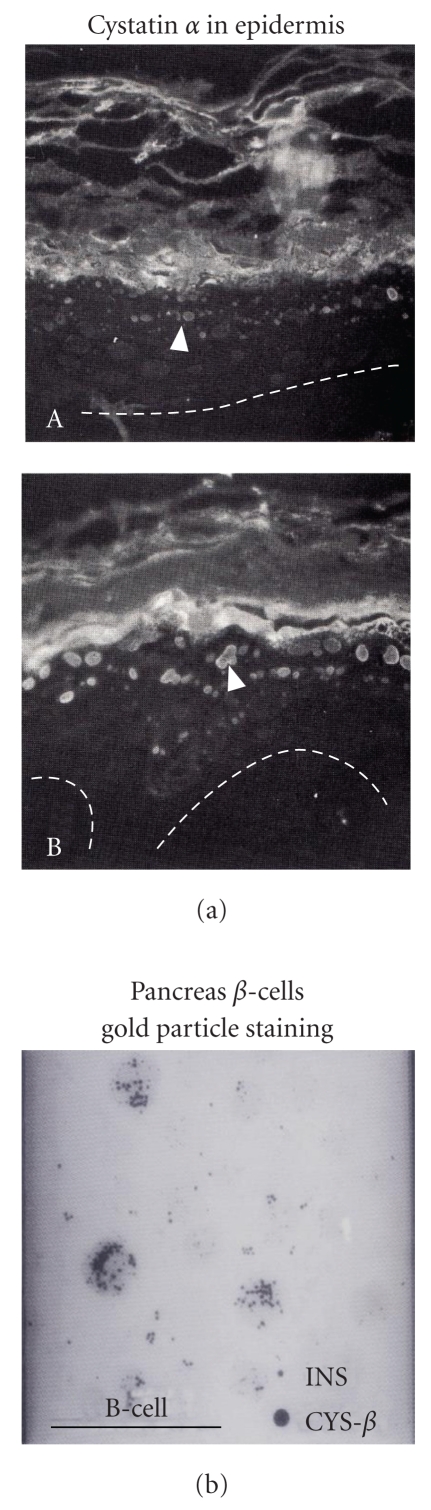
(a) Cystatin *α* (phosphorylated cystatin *α*) is localized in keratohyalin granules, (B) the treatment of protein C kinase inhibitor H7. No targeting of cystatin *α* in granules was observed. (A), (b) The localization of cystatin *β*. Cystatin *β* is localized and co-localized with insulin secretary granules.

When skin was stained by immunohistochemistry using anticystatin *α* antibody, only the cornified-envelope of skin was stained [16]. The sphingosine treatment to new born skin resulted in the strong suppression of the targeting of cystatin *α* into the cornified envelope. The sphingosine is a powerful inhibitor for protein kinase C. As shown in Figures 7(a) and 7(b), threonine residue in near C-terminus (the 92th threonine in Figure 9 shows) of cystatin *α* is phosphorylated by protein kinase C, and the phosphorglated cystatin *α* is incorporated into cornified envelope. The participation of protein kinase C in the phosphorylation of cystatin *α* was also confirmed using specific inhibitor of protein kinase C, (Hidaka H-7) inhibited the incorporation of P^32^ into cystatin *α*.

The phosphorylated cystatin *α* was incorporated into skin cornified envelop. In the envelop, the phosphorylated cystatin *α* was conjugated with filaggrin-linker sediment peptide which is rich in glutamine residues, mediated by epidermal transglutaminase in the presence of calcium, and yield a high-molecular weight protein (skin fiber), as shown in Figure 10.

The phosphorylated cystatin *α* showed strong inhibition to cathepsin L, but not to the cathepsin B and H, it also showed strong inhibition to the bacterial cysteine proteases and virus cathepsins. Therefore, cystatin *α* and phosphorylated cystatin *α*  showed strong protection from infections of bacteria or viruses in skin.

#### 3.1.2. Cystatin *β* [26, 27]

**Figure 8:**
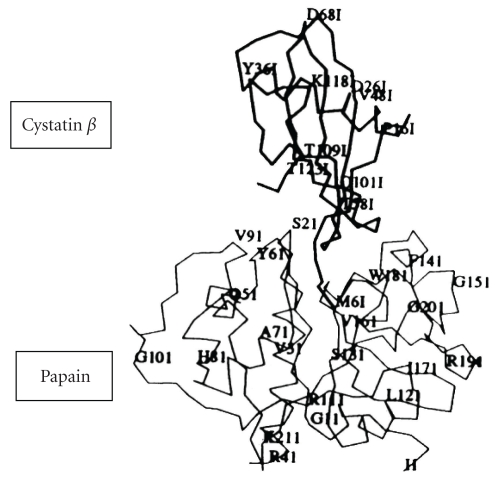
X-ray crystallography structure-binding complex between cystatin *β* and papain.

**Figure 9:**
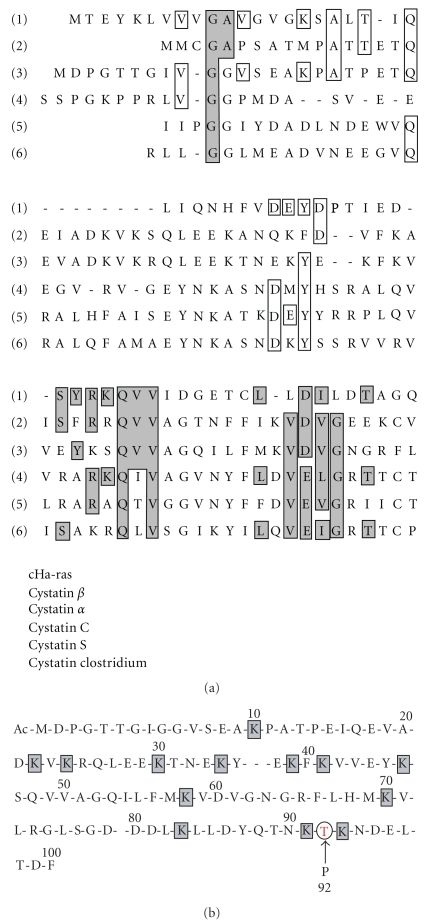
(a) Sequence homology of cystatin family. (b) Phosphorylated cystatin a. The targeting signal of incorporation of P-cystatin *α* into cornified envelop. (The 92th-Theonine residue).

**Figure 10:**
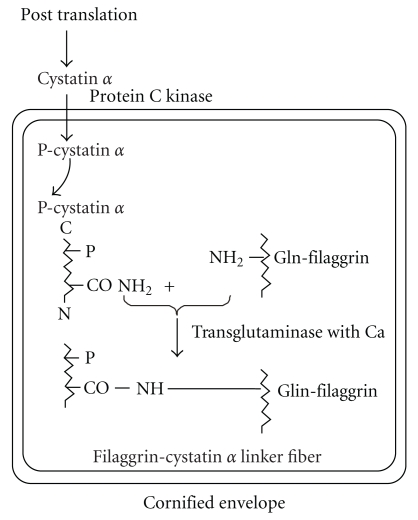
Post-translational modifications of cystatin *α*-phosphorylation, translocation, and filaggrin conjugation by transglutaminase (with Ca) to make filaggrin-Cysteine *α*-Linker Fiber.

Post-translational covalent modification of cystatin *β*  and the changing of their inhibitory activities. Cystatin *β* is located ubiquitously in lysosomes of various organs. The third position of the N-termius-cysteine residue of cystatin *β* reacts with glutathione to form a mixed disulfate complex and also is able to make dimmer by the disulfate bond. Those are inactive forms. As Figure 8 shows, N-terminus of cystatins was inserted into the substrate binding pocket of cathepsins, the glutathionated or the dimmer form were unable to insert into the binding pocket of cathepsins*.* The coefficient of oxidized or reduced forms of glutathiones regulates the inhibitory activity of cystatin *β*. Therefore, the activities of cathepsins are regulated by the intracellular redox potentials in spite of the changes of the inhibitory activities of cystatin *β*.

 As Figure 7(b) shows, using *β*-cell of pancreas, the cystatin *β* is located in insulin secretion particles, therefore, cystatin *β* is secreted with insulin in the pancreas.

## Figures and Tables

**Figure 3:**
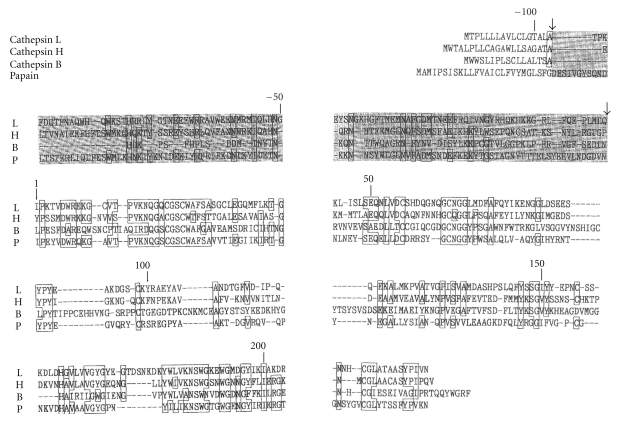
Comparison of total amino acids sequences of cathepsins L, H, B, and Papain. (Common sequences are the entrenched circles) and (Dark-background parts are pro-parts).

**Figure 6:**
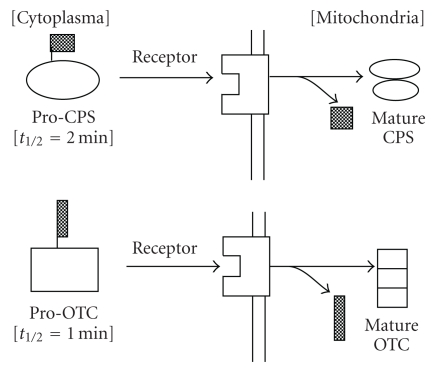
Roles of pro-part as a signal peptide of mitochondrial specific target receptors during the maturation of CPS and OTC in mitochondria.

**Figure 11:**
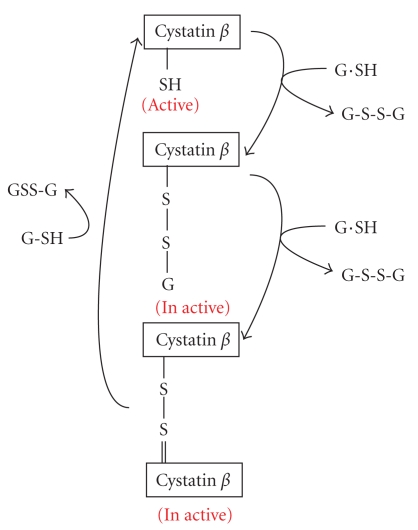
Functional modification of cystatin *β* with glutathione by disulfate-bond formation.

**Table 1:** Half-lives and content of cathepsins by E-64 administrations.

	Half-lives (h)		Content in lysosomes	
			(mg/mg protein)	
Cathepsin B	14	50	330	920
Cathepsin H	14	30	280	600
Cathepsin L	24	100	105	510
	(Control)	(E-64)	(Control)	(E-64)

**Table 2:** Secretion profiles of cathepsins from various cells.

Macrophages	Osteoclast	Fibroblast	Cancer cells
Secretion as proforms	Secretion as mature active forms	Nonsecrete	Secreted in metastasis cells
—	Stimulated by PTH Bone collagen degradation	I cell diseases Secreted into serum as mature forms	Nonmetastasis cells not secreted

**Table 3:** Composition of bond sugars in cathepsin B [14].

$\left(\begin{array}{c} 38\;\text{Asn}\\ \text{binding} \end{array}\right)$	Man^a-1^→6 Man^b1^→4GlcNAcb1→4GlcNAc Fue a1→6	26%
$\left(\begin{array}{c} 111\;\text{Asn}\\ \text{binding} \end{array}\right)$	Man^a-1^→6 Man^b1^→4GlcNAcb1→4GlcNAc	10%
	Man^a-1^ →6 Man^b1^→4GlcNAcb1→4GlcNAc	3.3%
	Man^a-1^→3 Man^b1^→4GlcNAcb1→4GlcNAc	

**Table 4:** Contribution of cathepsin L or B on bone resorption.

Inhibitors (10^−6^M)		Cathepsin L	Cathepsin B	Bone resorption	
Control		100	100	100	%
Pepstatin	−	103	104	104	%
CA-074	−	100	2.2	94	%
E-64	+	10	2	40	%
Cystatin *α*	+	3.1	4.3	18	%
Chymostatin	+	9.8	101	6	%
CA-148	+	0	100	0	%

**Table 5:** Organ distributions of cystatin *α* and *β* [16].

Organs	Cystatin *α*	Cystatin *β*
	(ng/mg protein)	
Epidermis	2800	320
Kidney	0.5	200
Liver	0.2	230
Brain	0.3	310
Muscle	0.3	76
Margen	820	420
